# Enhancing trash classification in smart cities using federated deep learning

**DOI:** 10.1038/s41598-024-62003-4

**Published:** 2024-05-23

**Authors:** Haroon Ahmed Khan, Syed Saud Naqvi, Abeer A. K. Alharbi, Salihah Alotaibi, Mohammed Alkhathami

**Affiliations:** 1 Department of Electrical and Computer Engineering, COMSATS University Islamabad, Islamabad, 45550 Pakistan; 2 Information Systems Department, College of Computer and Information Sciences, Imam Mohammad Ibn Saud Islamic University (IMSIU), Riyadh, 11432 Saudi Arabia

**Keywords:** Solid waste management, Recycling, Classification, Convolutional neural network, Deep neural network, Computer science, Information technology

## Abstract

Efficient Waste management plays a crucial role to ensure clean and green environment in the smart cities. This study investigates the critical role of efficient trash classification in achieving sustainable solid waste management within smart city environments. We conduct a comparative analysis of various trash classification methods utilizing deep learning models built on convolutional neural networks (CNNs). Leveraging the PyTorch open-source framework and the TrashBox dataset, we perform experiments involving ten unique deep neural network models. Our approach aims to maximize training accuracy. Through extensive experimentation, we observe the consistent superiority of the ResNext-101 model compared to others, achieving exceptional training, validation, and test accuracies. These findings illuminate the potential of CNN-based techniques in significantly advancing trash classification for optimized solid waste management within smart city initiatives. Lastly, this study presents a distributed framework based on federated learning that can be used to optimize the performance of a combination of CNN models for trash detection.

## Introduction

Urban landscapes are growing and evolving rapidly. With growing size and population, it is becoming quite challenging to manage the scale of waste management. While recycling is being preached and practiced to varying degrees around the world, the growing hazard of unmanageable waste has reinvigorated research into new and maintainable methods to recycle waste^1^. Delivering approaches to sustainable recycling is considered one of the leading challenges facing the environment and the economy today. The need of the hour are holistic policies and approaches to ensure recyclability and recycling. These policies would serve to reduce the burden and reliance on natural resources, a decrease in greenhouse gas emissions and a lower carbon footprint^1,2^. This policy should be based on a vision of a circular economy, where products are designed to be recycled, and where materials are continuously reused and repurposed^2^. Comprehensive realization of such a policy would be impossible without employing the latest tools and technologies that can be leveraged to maximize every aspect of waste management and recycling.

**Figure 1:**
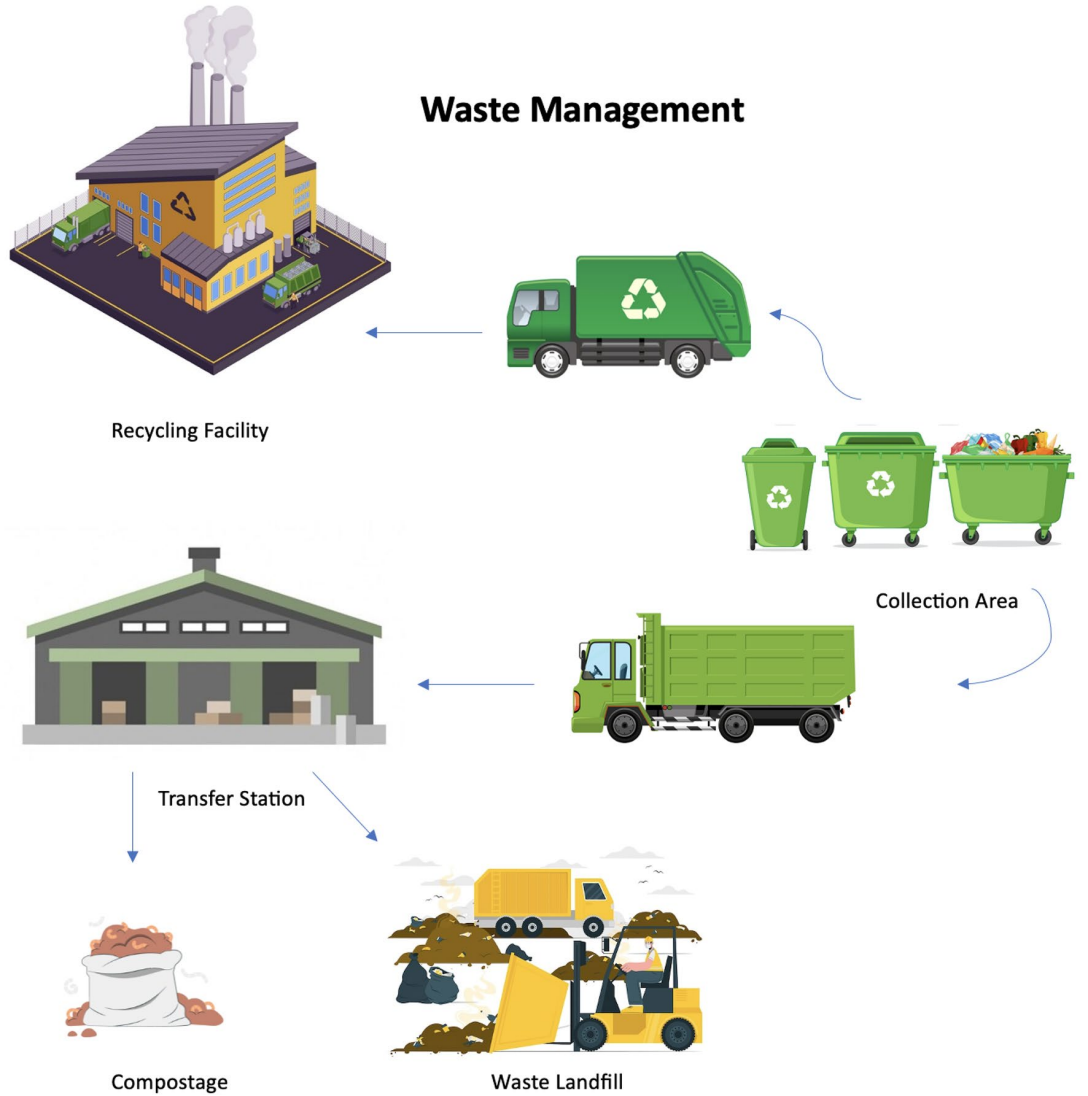
Waste management process.

The term waste management can have a wide range of meanings. Typically, all policies and procedures adopted to collect, manage, dispose of and reuse waste would fall in the purview of waste management. It should have comprehensive oversight on tactics to reduce waste, manage its production, its transport, and ultimate disposal. Disposal may include recycling, composting, incineration, landfilling, waste-to-energy conversion etc, as illustrated in Fig. 1. Waste management would entail optimizing the use these methods in accordance with the nature of the economy, nature of the society and nature of the waste, with an emphasis towards reduction, reuse and recycling of waste.

Recycled materials hold significant economic value, creating new markets and job opportunities in collection, processing, and manufacturing sectors. Diverting them from landfills translates to reduced waste disposal costs for municipalities, freeing up resources for crucial urban development projects^2^. Moreover, investments in cutting-edge sorting technologies and infrastructure fuel innovation in areas like artificial intelligence and robotics, leading to more efficient and cost-effective solutions^3^. By prioritizing and actively promoting effective sorting practices, smart cities can unlock a path towards a more sustainable and resource-efficient future. This approach benefits not only the environment but also the economy and the well-being of urban communities.

As the world becomes more environmentally conscious, there is a growing emphasis on recycling a wide range of materials. Despite encountering obstacles, global patterns suggest a notable increase in the gathering and treatment of recyclable materials spanning different categories. In^4^, a growing awareness of responsible medicine waste management is suggested. The study highlights an increase in regulations and collection systems. The authors in^5^ highlights the widespread reluctance to adopt environmentally sustainable methods for healthcare waste disposal on a large scale, citing cost, accessibility, and feasibility as key barriers. There remains a lack of a universally scalable green solution. Furthermore, the review underscores that the global health implications of healthcare waste disposal methods tend to vary based on the level of development of the country.

Similar to healthcare waste, the management of electronic waste (e-waste) has garnered significant attention globally. In^5^, proposals for new regulations and increased enterprise collaboration have been advocated. In light of these developments, it is imperative to conduct an overview to assess the current status and explore potential new solutions and challenges concerning e-waste management in China. It has been reported that both central and local governments in China have made substantial endeavors to enhance e-waste management^6^. The presence of heavy metals in municipal solid waste underscores the necessity for effective waste management strategies due to limitations on its beneficial use or disposal. Understanding the occurrence of heavy metals in municipal solid waste is pivotal for refining waste handling practices. Compared to other pollutants, heavy metals 62 are extremely toxic and can contaminate soil, water, and air. They pose a serious risks to the 63 environment and health of all manner of living things. Physicochemical-fractionation has been shown to be a potentially viable approach to manage heavy metal waste by facilitating the separation and concentration of heavy metals from waste streams^7^. However, in the context of a city or municipality, using fractionation poses numerous challenges, as typical waste material is not as controlled or consistent as industrial raw materials. These approaches are not viable unless the chemical composition of the various articles of waste can reliably be determined. It would require stringent approaches to source control, waste sorting and regulatory compliance monitoring for fractionation to be integrated in existing waste management frameworks^7^.

While the importance of reuse and recycling is well established, recent literature has determined that optimizing the sorting and separation of waste at its source would have the most significant impact in the management and recycling potential of waste^8^. Such approaches would also serve to minimize contamination. This would entail the segregation of recyclable materials at their point of origin, such as households or businesses, before they enter the waste stream. By promoting and enforcing stringent communal waste sorting practices, recycling rates can be enhanced to achieve a net reduction in waste disposal. This would contribute to the conservation of resources and would reduce the load on rapidly growing landfills^8^.

Conventional manual sorting methods are costly, hard to scale and impractical when it comes to widespread applicability. Consequently, the demand for automated waste segregation systems is growing significantly^9^. Many existing automation techniques are simplistic mechanical sorting devices that do not use smart computerized control systems. However, many approaches have recently started to incorporate smart computational platforms to automate the sorting process. Sensors are placed on the trash bins and data is transmitted to the cloud servers using cellular wireless communications. Many of these approaches use artificial intelligence (AI), machine learning (ML) and neural networks to make these platforms smart. This research presents a comparative study of 10 waste classification methods that visually identify different types and classes of waste using deep neural networks (DNNs). This research tested the models using the open source PyTorch framework. The dataset used to test all 10 models was the TrashBox dataset^9^. A secondary outcome of this research was to introduce techniques to enhance the training accuracy of these models. Any model that can accurately detect and identify various classes of waste materials at a high speed has the potential to contribute to the development of a revolutionary waste sorting system.

## Related work

To overcome trash sorting challenge, novel approaches leveraging deep neural models trained on diverse trash image datasets have emerged. This section offers an overview of research endeavors in trash detection and classification.

TrashNet^10^, a dataset featuring 2527 images across six trash classes was developed. Despite its limited size, TrashNet facilitated experiments with CNN and SVM models, with the SVM model achieving the highest accuracy at 63%. Another dataset, TACO (Trash Annotations in Context)^11^, presents images of trash objects found in various settings but with fewer annotations compared to TrashNet. To overcome the issues of both TrashNet and TACO datasets, the authors in^9^ developed a TrashBox dataset having more images and classes as compared to its counterparts.

Several studies have delved into machine and deep learning models for waste classification. The authors in^12^ utilized the ResNet-50 transfer learning model on TrashNet, achieving 87% accuracy with a multi-class SVM approach. Similarly, the authors in^13^ employed a CNN model with the Inception-v3 transfer learning model on the Plastic Model Detection dataset, reporting 92.5% accuracy. In^14^, the authors developed ScrapNet, based on the EfficientNet transfer learning model, achieving 98% accuracy on TrashNet and 92.87% on a new dataset. In^15^, a waste classification method is proposed using a multilayer hybrid CNN, achieving a classification accuracy of up to 92.6% on TrashNet.

The performance of transfer learning models trained using the TrashBox dataset to evaluate their adaptability. Impressively, these models demonstrated an accuracy rate of 98.47%. Moreover, a pioneering deep learning framework integrating quantum transfer learning methodologies is also explored in^9^. It is important to mention that in^16^ the large-scale TrashBox dataset is also suggested as to be a promising choice for training CNNs in waste classification. RecycleNet^17^ introduces an optimized deep learning architecture tailored for classifying recyclable objects, utilizing the TrashNet dataset. Hybrid deep learning approaches, as explored by^18^ and^19^, investigate the impact of image features on classification performance, demonstrating the effectiveness of Multilayer Hybrid Systems and CNNs. Medical waste classification receives attention with ResNeXt models^20^, while DSCR-Net^20^ and studies like^21^ focus on open-source datasets and single-object identification, respectively.

Furthermore, studies investigate deep learning-based methods for garbage classification, with models like GCNet^22^ and DSCAM^23^ demonstrating high accuracy rates. Practical applications such as AlphaTrash^24^ offer automated sorting solutions, addressing the need for efficient waste management systems. Augmentation techniques for addressing data scarcity are explored by^25^, while metadata-based approaches^26^ and robotics-driven classification^27^ contribute to the research landscape. Moreover, studies utilizing CNN models^28,29^ underline the ongoing efforts to improve trash classification methodologies and their practical implications in waste management practices. Table 1 provides a comparison of some of the existing trash classification studies.

**Table 1:** Trash classification in existing literature.

Model	Methodology	Key findings	Contributions
RecycleNet^17^	Deep learning architecture tailored for trash classification using TrashNet dataset	Achieved high accuracy in classifying recyclable objects	Improved trash classification in waste management
Hybrid approaches^18,19^	Investigation of Multilayer Hybrid Systems and CNNs	Effectiveness of hybrid models in trash classification	Enhanced understanding of image features’ impact
ResNeXt models^20^	Focus on medical waste classification	High accuracy in identifying medical waste types	Improved management of medical waste
DSCR-Net^20,21^	Utilization of open-source datasets and single-object identification	Development of open-source datasets and identification techniques	Enhanced accessibility and efficiency in trash classification
GCNet^22^, DSCAM^23^	Deep learning-based trash classification with high accuracy rates	Demonstrated effectiveness of deep learning models	Improved accuracy and reliability in trash classification
AlphaTrash^24^	Automated sorting solutions for efficient waste management	Offered practical solutions for automated trash sorting	Improved efficiency and effectiveness in waste management
Augmentation techniques^25^	Exploration of data augmentation techniques to address data scarcity	Effectiveness of augmentation methods in improving model performance	Enhanced robustness and reliability of trash classification models
Metadata-based approaches^26^	Utilization of metadata for trash classification	Improved classification accuracy based on metadata information	Enhanced understanding and utilization of metadata in trash classification
Robotics-driven classification^27^	Trash classification using robotics-driven approaches	Application of robotics for trash classification tasks	Enhanced automation and efficiency in trash classification
CNN models^28,29^	Utilization of CNNs for trash classification	Ongoing efforts to improve trash classification methodologies	Continued advancement in trash classification techniques

## TrashBox dataset overview

To fill the significant gap observed in existing trash detection and classification datasets, researchers developed a new dataset called TrashBox^9^. This initiative stemmed from a critical assessment of prevailing benchmark datasets like TrashNet and TACO, revealing notable deficiencies in image frequency and representation of key waste types.

**Figure 2:**
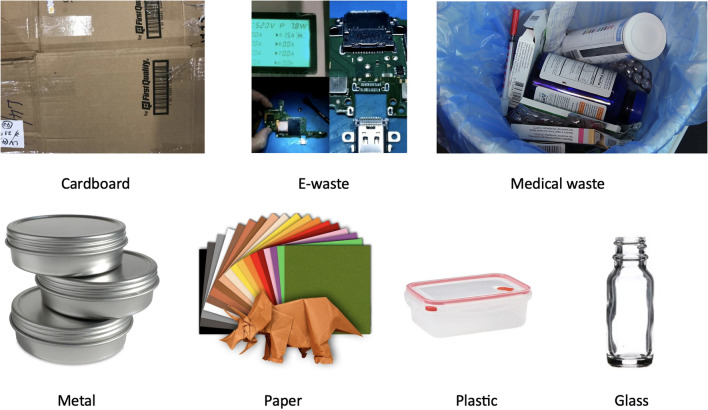
Snapshots from TrashBox dataset for different classes.

The analysis underscored the limited availability of images necessary for precise trash object classification, a concern acknowledged by the datasets’ own authors. Additionally, the absence of crucial waste categories like e-waste and medical waste highlighted the urgent need to address this gap, given their significance in contemporary waste management practices. In response, TrashBox was thoroughly constructed to encompass a diverse range of trash objects commonly encountered across various environments. The dataset comprises seven primary classes-medical waste, e-waste, glass, plastic, cardboard, paper, and metal-each carefully curated to ensure comprehensive coverage of trash types encountered in real-world scenarios. Some images of the TrashBox dataset for different classes are shown in Fig. 2. Furthermore, these classes are subdivided into subclasses to enable finer distinctions between trash objects and support nuanced research and analysis in the field.

The TrashBox dataset comprises various trash classes and their corresponding sub-classes, along with the number of images available for each category is shown in Table 2.

**Table 2:** Details of the TrashBox dataset.

Trash classes	Sub-classes	No. of images	Total
Cardboard	Assorted cardboard objects	2414	2414
E-waste	Electric wires	568	2883
	Laptops and smartphones	774	
	Small appliances	926	
	Electrical chips	615	
Metal	Beverage cans	1000	2586
	Construction scrap	539	
	Spray cans	500	
	Metal containers	505	
	Miscellaneous metal	42	
Plastic	Plastic bags	504	2669
	Plastic bottles	571	
	Plastic containers	580	
	Plastic cups	507	
	Cigarette butts	507	
Paper	Tetra pak	794	2695
	News paper	200	
	Paper cups	639	
	Other paper objects	1062	
Glass	Assorted glass objects	2528	2528

The development of the TrashBox dataset required a substantial effort. The first step mandated a meticulous and systematic approach to collect data, i.e. large volumes of photographs of trash. Deep learning algorithms typically require tens of thousands of images before training becomes feasible. Various tools were used to download images from various reputable online platforms. However, raw images cannot be used directly in a deep learning context. Numerous pre-processing tasks have to be performed to bring all images to a consistent standard and quality. These processes may involve resizing, calibrating aspect ratios, cropping, quality adjustments, noise removal and finally labelling. Labelling is the process of identifying the various classes of trash in the images that will be used to train the deep networks. To date, TrashBox serves as one of the most important repositories of a large number of diverse images of trash, immensely aiding researchers, and practitioners in the field of trash detection and classification.

## CNN-based classification techniques

In this section, we briefly explain CNN-based classification models that are commonly used in the literature. In this study, we use these DNN models to classify the TrashBox dataset.

### GoogLeNet

GoogLeNet^38^, also referred to as Inception-v1, stands out as a CNN architecture developed by Google. It is distinguished by its unique “Inception” modules, comprising multiple convolutional layers of varying sizes and types. A notable feature of GoogLeNet was its capability to achieve high accuracy in image classification tasks while ensuring computational efficiency, compared to other models that were available at the time of its release. GoogLeNet demonstrated its ability to capture features of varying scales and complexities to analyse images. Their efficiency was attributed to the smart resource utilization by the underlying inception modules. GoogLeNet further introduced concepts such as dimensionality reduction and parallel feature extraction, that are a staple of all modern CNNs today. While GoogLeNet was primarily designed for visual computing tasks such as object detection and image segmentation etc., many researchers were able to show case its applicability to other tasks outside these domains. GoogLeNet contributed significantly to the popularity of CNNs in a wide range of applications^31^.

### ResNet model

The deeper neural networks get, the harder and more time consuming it got to train them. ResNet (Residual Network) solved this problem by introducing the concept of using shortcuts known as “skip connections” for the feedforward operation. The idea is to allow for the skipping or bypassing of certain CNN layers, while reusing the activation functions of the previous layers^32^. This is made possible by the use of residual mapping, where layers learn from it instead of the underlying mapping. The skip connections perform identity mapping and are added to the outputs of the stacked layers.CNNs with residual mapping mechanism have proven to be easier to optimize, compared models without residual mapping. The authors of ResNet have demonstrated that they can incorporate all these feathers without adding any added computational complexity^32^. Furthermore, ResNets were the first CNNs that were able to support hundreds of thousands of convolutional layers. The innovations introduced in ResNet made it a cutting-edge model of its time allowing the application of deep CNNs in a real-world context. ResNets still are a compelling models when it comes to image classification tasks.

### ResNeXt model

The ResNeXt model^33^ is based n the ResNet model that has introduced a additional dimension of cardinality, which is the introduction of a number of parallel transformation paths within a network layer. Within this architecture, a network module conducts a series of transformations on low-dimensional embeddings, and then integrating their outputs through summation. This design facilitates scalability to accommodate numerous transformations without necessitating specialized designs. Empirical evidence demonstrates that the model outperforms the original ResNet module, even while preserving computational complexity and model size. It has broad applicability in both visual and non-visual recognition tasks.

### MobileNetV2

MobileNetV1 was developed in 2017 to cater to the unique needs of embedded systems and mobile phones. MobileNetV2 advances upon the original MobileNet by introducing several notable improvements. MobileNetV2 adopts inverted residual blocks with linear bottlenecks, enabling optimal resource utilization while upholding model accuracy. Moreover, it incorporates depth-wise separable convolutions to reduce parameters and computational costs without compromising spatial information capture. MobileNetV2 is a signifcant improvement over MobileNetV1 when it comes to both accuracy and latency. These improvements can be attributed to innovative architectural features such as linear bottlenecks, residual connections, and improved skip connections^34^. A large body of literature has shown that MobileNetV2 consistently performs a wide variety of image processing tasks pretty well, especially in the context of a mobile or embedded use-case. This would suggest that MobileNetV2 is a robust and efficient choice for deploying deep CNN models on devices with constrained resources, making it an excellent candidate when it comes to applications like mobile phone-based image recognition or object detection.

### MobileNetV3

The latest version of MobileNet, known as MobileNetV3, comes in two variants: small and large, developed by the project’s contributors^35^. The small variant prioritizes efficiency, making it ideal for devices with limited processing power. It achieves compactness through structural improvements in its underlying convolutional neural network architecture. This makes it a suitable option for tasks like image categorization and identifying objects in images. Conversely, the large variant prioritizes accuracy and performance, making it better suited for complex image processing and visual computing applications. Both variants utilize innovative features like efficient attention mechanisms and advanced optimization techniques, expanding their applicability across various domains.

### ShuffleNetV2

ShuffleNetV2^36^ is a CNN-based model. ShuffleNetV2 prioritizes real-time responses 237 in image processing tasks, significantly improving upon its predecessor, ShuffleNetV1. 238 This version incorporates a unique channel split operation within its blocks, enabling it to 239 handle dependencies between channels more efficiently compared to the earlier version. 240 Additionally, the processing pipeline has been restructured, strategically positioning the 241 channel shuffle operation deeper within the block architecture. While this did not required 242 any addition of a component in the pipeline, it lead to improved performance of the network 243 over V1. The V2 has additionally incorporated techniques like feature map reorganization 244 and channel shuffling to improve feature representation and extraction, which makes it 245 more robust. All these considerations makes ShuffleNetV2 an excellent candidate for a 246 use-case where real-time response is of critical importance.

## Results

In this section the performance of the models that we tested using the TrashBox dataset will presented and analyzed numerically. This section is divided into two parts, where in the first part we explain the experimental setup and in the second part we discuss the classification results obtained using different deep neural network models.

### Experimental setup

To perform the task of classification of trash within the TrashBox dataset, we employ DNNs utilizing the PyTorch open-source platform. Specifically, we utilize a set of 10 DNNs for classification purposes. These models undergo training utilizing the Nvidia GF GTX 1080 GPU with 64GB of RAM. The experimentation phase for all the models is run for 30 epochs. The division of data into training, validation and test set are according to the original TrashBox dataset^9^.

### Performance comparison of various DNNs

Upon analyzing the results obtained from various DNN models applied to classify the TrashBox dataset, it is evident from Tables 3 and 4 that ResNeXt-101 and ResNeXt-50 consistently outperform other models in terms of accuracy across training, validation, and test datasets. ResNeXt-101 achieves the highest test accuracy of 89.62$\%$ and the highest test F1 score of 89.66$\%$, indicating its superior performance in classifying the dataset. ResNeXt-50 also exhibits strong performance with a test accuracy of 88.68$\%$ and a test F1 score of 87.01$\%$.

ShuffleNetV2, despite demonstrating lower training accuracy compared to ResNeXt models, surprisingly shows comparable validation and test accuracies, suggesting potential overfitting in the ResNeXt models. However, its test F1 score of 83.11$\%$ indicates slightly lower performance in capturing dataset nuances compared to ResNeXt models. Among ResNet models, ResNet-34 shows decent performance with a test accuracy of 87.07$\%$ and a test F1 score of 86.86$\%$, while ResNet-50 and ResNet-101 perform slightly lower, albeit still commendable.

MobileNetV2 and MobileNetV3-Large display relatively good performance, although their test accuracies and F1 scores fall short of ResNeXt and ResNet models. MobileNetV3-Small exhibits the lowest performance among MobileNet models, suggesting potential underfitting due to its lower complexity. GoogLeNet achieves competitive performance with a test accuracy of 86.18$\%$ and a test F1 score of 85.71$\%$, positioning it among the top-performing models, although slightly below ResNeXt models.

From Tables 3 and 4 ResNeXt-101 emerges as the superior model due to its consistently high accuracy and F1 score across all datasets. ResNeXt-50 also performs well, albeit slightly lower than ResNeXt-101. ShuffleNetV2 shows promise despite its lower complexity. This shows the potential applicability of ShuffleNetV2 in an embedded system or mobile-based platform. The most crucial aspect to consider in this study is the balance between model complexity and performance. The contrast between ResNeXt and MobileNet models can be observed to see that models with higher complexity often leads to better results but requires more computational resources and vice-versa.

**Table 3:** Accuracy (in percentage $\%$) with various DNN-based classification schemes for TrashBox dataset.

Technique	Accuracy		
	Training	Validation	Test
ResNeXt-101^33^	**97.86**	**97.15**	**89.62**
ResNeXt-50^33^	97.66	95.18	88.68
ShuffleNetV2^36^	88.48	89.99	82.91
ResNet-34^32^	96.31	95.25	87.07
ResNet-50^32^	96.98	95.06	87.62
ResNet-101^32^	97.31	95.82	87.24
MobileNetV2^37^	94.55	94.11	86.24
MobileNetV3-Large^35^	92.74	93.28	85.13
MobileNetV3-Small^35^	85.60	87.96	81.80
GoogLeNet^38^	93.41	94.23	86.18

Significant values are given in bold.

**Table 4:** $F_{1}$ score (in percentage $\%$) with various DNN-based classification schemes for TrashBox dataset.

Technique	${F_{1}}$ Score
ResNeXt-101^33^	**89.66**
ResNeXt-50^33^	87.01
ShuffleNetV2^36^	83.11
ResNet-34^32^	86.86
ResNet-50^32^	87.69
ResNet-101^32^	87.03
MobileNetV2^37^	86.06
MobileNetV3-Large^35^	85.27
MobileNetV3-Small^35^	82.04
GoogLeNet^38^	85.71

Significant values are given in bold.

**Figure 3:**
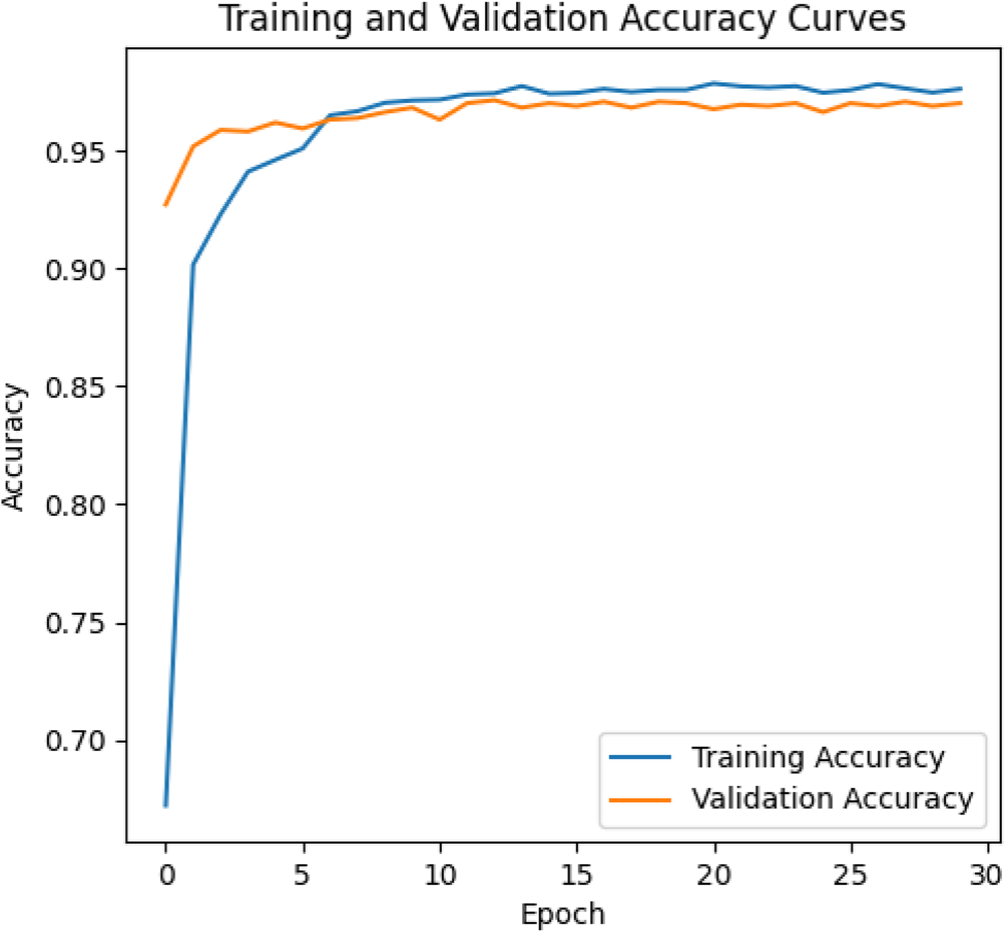
Training accuracy and validation accuracy for ResNet-101 DNN.

The training and validation accuracy curves for ResNeXt-101, as displayed in Fig. 3, provide key information about its learning process and ability to perform well on new data. The model achieves a training accuracy of 97.86% and a validation accuracy of 97.15%. These high values indicate that ResNeXt-101 learns effectively from the training data and can generalize well to unseen examples. In simpler terms, the model effectively captures the underlying patterns within the data without memorizing specific details, reducing the risk of overfitting.

**Figure 4:**
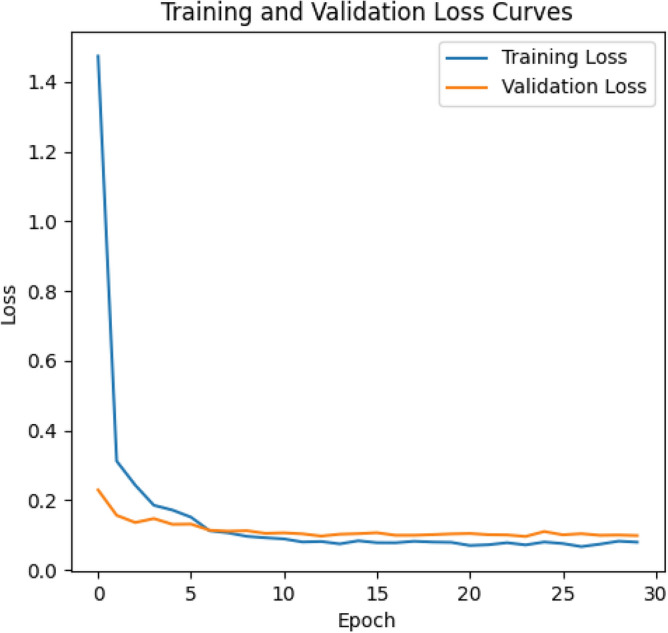
Training loss and validation loss for ResNet-101 DNN.

The training loss and validation loss curves for ResNeXt-101 can be seen in Fig. 4. The figures gives us some insight into its training dynamics and generalization capacity to unseen data. Throughout the training process, both training and validation loss curves stabilize at approximately 0.1, indicating the model’s convergence to a consistent performance level. This stabilization suggests that the model has grasped the underlying data patterns effectively and has reached a point where further training yields marginal improvements. A noteworthy observation is the consistent trend of the training loss curve dipping below the validation loss curve post the 5th epoch, persisting throughout the training duration. While this hints at potential overfitting to the training data, the narrow gap between the two curves suggests minimal overfitting.

**Figure 5:**
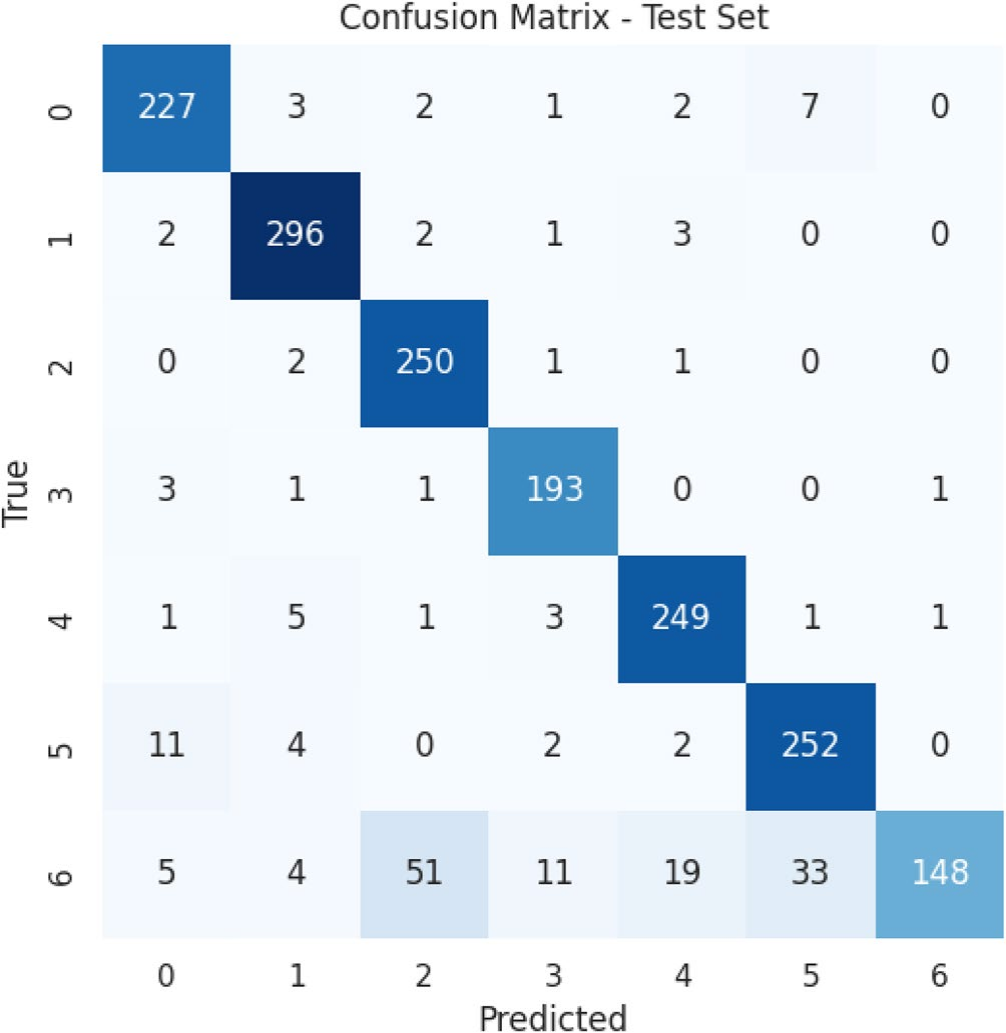
Confusion matrix for ResNet-101 DNN.

The provided confusion matrix in Fig. 5 offers a detailed breakdown of a ResNeXt-101 classification model’s performance across seven distinct waste classes: cardboard, e-waste, glass, medical, metal, paper, and plastic. Each row represents the actual class labels, while each column signifies the predicted class labels. The numerical values within the matrix indicate the count of instances classified into each respective class. For example, when examining the row corresponding to cardboard (Class0), it reveals that the majority of cardboard instances (227) were accurately classified as Class0. However, there are some misclassifications, such as a small number of instances being erroneously labeled as glass (2), medical (1), metal (2), paper (7), and no instances being misclassified as plastic. By scrutinizing other rows, we can assess the accuracy and misclassification rates for each waste class. For instance, e-waste (Class1) demonstrates high accuracy with minimal misclassifications in other classes. Conversely, glass (Class2) exhibits relatively favorable performance with limited misclassifications.

**Table 5:** Model complexities for the TrashBox dataset.

Technique	Computational complexity (GMac)	Number of parameters (M)	Model size (MB)
ResNeXt50	33.53	25.04	95.94
ResNet101	61.56	44.56	170.68
ResNet50	32.33	25.57	97.89
ResNet34	28.82	21.8	83.33
MobileNetV3-Small	232.6	2.54	9.83
MobileNetV3-Large	894.64	5.48	21.11
MobileNetV2	1.25	2.23	8.75
GoogLeNet	11.83	6.63	25.52
ResNext	31.14	83.47	319.46

The analysis of different techniques based on their complexities in Table 5 reveals varying trade-offs in computational resources and model sizes. In the view of this study, ResNeXt50 demonstrates the most balanced approach amongst all models that were compared. It presents a relatively moderate computational complexity and model size, making it adaptable for a variety of applications. The higher the computational complexity and requirement for the model size is, the more resource-intensive the architecture becomes. This is where the debate for a balance between accuracy and resource requirements arises. Considering the performance of ResNeXt50, ResNet101 exhibits slightly lower computational complexity and model size requirement, that is reflected in its slightly lower performance. ResNet50 strikes a middle ground, offering a reasonable compromise between complexity and performance, balancing efficiency, and accuracy. This downward trend of model complexity continues with ResNet34 all the way to MobileNet-based models, as can be seen in Table 5. As the name suggests, MobileNet models are quintessential light-weight models designed primarily for mobile devices, that are becoming increasingly popular for deployment on embedded systems and edge devices. Due to their relatively light architectures, they have low computational requirements, and consequently, low power requirements. However, they have been shown to work reasonably well, as is evident in this study as shown in Tables 3 and 4. To summarize, high performance corresponds to high computational requirements and large model size requirements. The choice of deployment of a model would be contingent on the criticality of performance versus the type of computational platform that is most suitable for deployment. This would vary from use-case to use-case and it would require a careful consideration of all associate factors before the most suitable model can be chosen.

## Proposal for a federated learning framework

The various machine learning models, discussed in the previous section, have proven track records when it comes to discriminating and classifying different kinds of objects in images and video frames. While they may perform very well in distinguishing one kind of visual artefact, they may not perform equally well for something else^39^. Each of the aforementioned model has its own strengths and weaknesses. Furthermore, their performances vary in terms of working conditions, i.e. quality and types of images they have to work with in real-time. An ideal approach would allow for models to cater to all kinds of environmental conditions while simultaneously leveraging the cumulative competence of several models. This is possible by using a distributed framework that is based on Federated Learning (FL). FL is an approach initially developed by Google. Federated learning comprises a framework that is able to maintain a federated (master) model in the cloud that periodically communicates with several models that are running remotely^38^. Each remote model matures during inference (with experience), that results in a change of their model parameters. The remote models periodically share their parameters with the federated server, that may use any number of algorithms to consolidate the parameters to devise a so-called “collective wisdom”. This collective wisdom is than broadcast back to all the remote sites so that they can benefit from the learning of all instances of the model.

**Figure 6:**
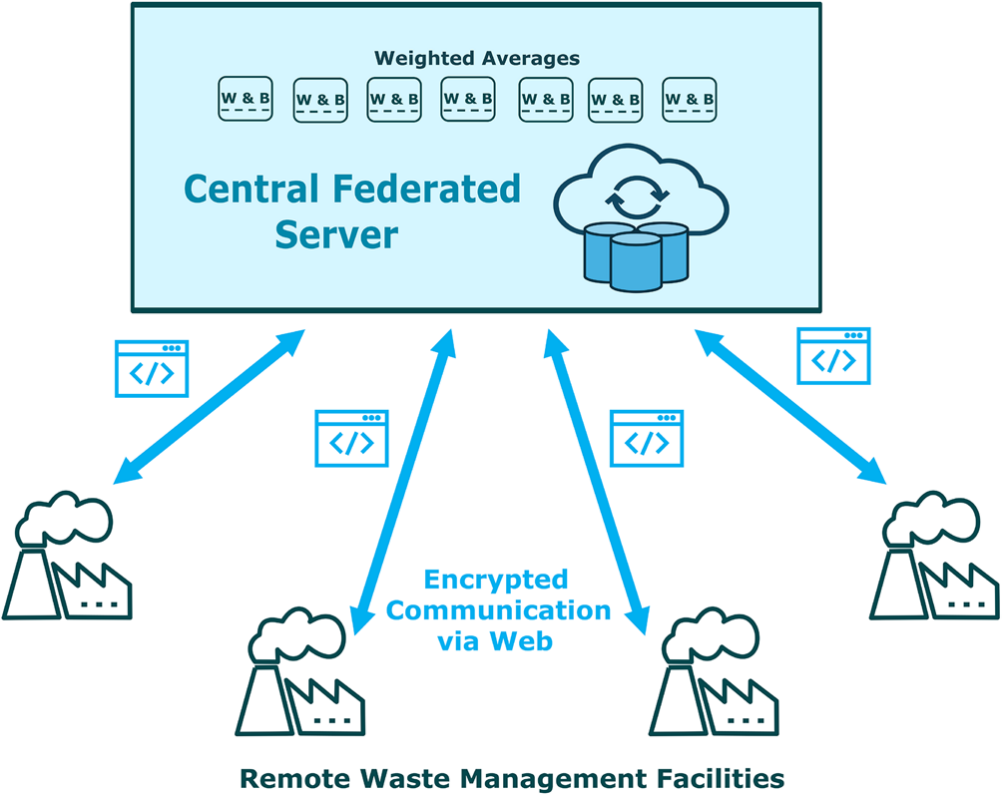
A federated network that receives updated weights and biases from all remotely-connected waste management facilities in an encrypted form, to consolidate the weighted averages to be broadcast back to all connected facilities.

This work proposes deployment of a federated framework for the visual detection of 7 different classes of trash at waste management facilities. Furthermore, it is proposed that 4 models be used that have been shown to work well with the TrashBox dataset, instead of using a single model alone. The performance of every model varies slightly compared to the identification class of trash, (i.e. metal, plastic, glass, etc.) and can therefore be used in tandem to get the best possible discrimination of trash overall. In the proposed framework, the federated server will compute the weighted average of model parameters, specifically weights (W) and biases (B). It will receive individual parameters for each model via encrypted communication channels from numerous waste management facilities. Each of the facility would be responsible to run those models in tandem, as shown in Fig. 6. In return, the federated server would broadcast the consolidated parameters back to each waste management facility.

**Figure 7:**
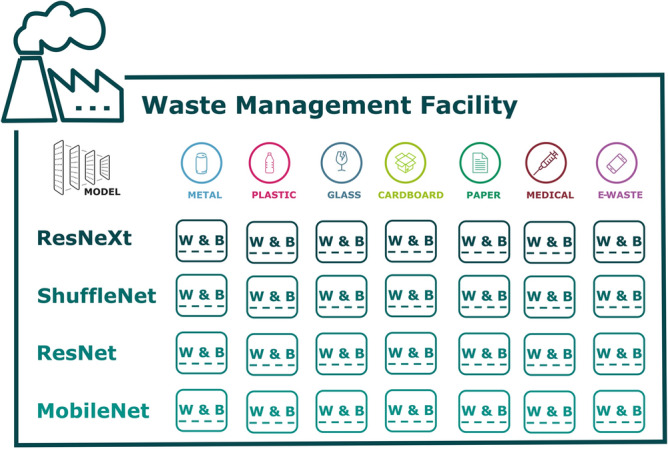
Each waste management facility should run 4 best performing detection models and optimize the parameters (weights and biases) in accordance to each class of trash, namely, metal plastic, glass, cardboard, paper, e-waste and medical waste.

Based on the benchmarks and tests conducted in this study, the four models proposed to run at each remote site are ResNeXt-101, ShuffleNetV2, ResNet-34 and MobileNetV3-Large respectively. All four models have been shown in this study to perform the task of detection of different classes of trash very well. Furthermore, all of these models can be configured to function as reinforcement learning (RL) agents. This means that they have the ability to improve and adapt their parameters in accordance to their real-time performance via reinforcement learning. This would allow each model to be optimized locally for each class of trash that is provided in the TrashBox dataset, as shown in Fig. 7. Each individual waste management site will be able to share its unique learning experience with other sites via the federated server.

## Conclusions

This study evaluated a variety of deep learning models to thoroughly test their efficacy in detecting and classifying different types of waste. The TrashBox dataset was used on all the models, that were tested on the same hardware platform. After rigorous testing, this study found that two models, namely, the ResNeXt-101 and ResNeXt-50 consistently outperformed other models both in training and post-inference validation. Of these two, ResNeXt-101 achieved the highest test accuracy of 89.62% and the highest test F1 score of 89.66%. ResNeXt-50 was comparable with an 88.68% accuracy and a test F1 score of 87.01%. ShuffleNetV2 showed potential with comparable validation and test accuracies with a slightly lower test F1 score. This may suggest a possible case of overfitting in the ResNeXt-based models.Among the ResNet-based models, ResNet-34 performs commendably well, followed by ResNet-50 and ResNet-101. They clearly outperform all the MobileNet-based models. This could be because MobileNet models are lightweight models built for relatively lower computational requirements resulting in potential underfitting.

In conclusion, while ResNeXt-101 stands out as the superior model, maintaining dependably high accuracy and F1 score, many of the other models were consistently accurate. making them viable to be applied in a trash-classification system. The key is balancing model complexity with the model performance. This trade-off was observed between ResNeXt and MobileNet models, where ResNeXt models were clearly optimal for performance while MobileNet models designed to run on low-end computational platforms. The ability to run on inexpensive embedded platforms may be a crucial consideration in a practical context. This comparative analysis emphasizes the importance of selecting the most suitable DNN model for effective trash classification, if used for the waste management of smart cities.

Lastly, this study has presented a proposal for a framework that is based on federated learning, that uses 4 image classification models at each waste management site. The concept is to integrate the model parameters of several such sites to a federated server, that would consolidate the model parameters, specifically, their weights and biases, to develop a global wisdom. Based on the tests and benchmarks performed in this study, the four most suitable models that should be deployed are ResNeXt-101, ShuffleNetV, ResNet-34 and MobileNetV3-Large respectively.

## Data Availability

All related data is available in the paper.
